# RACE, PRODUCTIVE ACTIVITIES, AND COGNITIVE FUNCTIONING IN THE HEALTH ABC STUDY

**DOI:** 10.1093/geroni/igae098.3699

**Published:** 2024-12-31

**Authors:** Ronica Rooks, Mirza Ishrat Noor, Elizabeth Vasquez

**Affiliations:** University of Colorado Denver, Denver, Colorado, United States; University at Albany, Albany, New York, United States; University at Albany, SUNY, Rensselaer, New York, United States

## Abstract

Productive activities (working and volunteering) promote older adults’ purpose in life, physical activity, and social support and may improve cognitive functioning. We hypothesized older adults working or volunteering will have higher cognition, and each productive activity will attenuate the relationship between race and cognition. We used the Health ABC study, a cohort of community-dwelling, well-functioning Black (48.4%) and White (51.6%) older adults aged 70-79 in year 1 (n=2,996). Our linear mixed-effects models included race, working or volunteering, gender, age, education, smoking, the 400-meter walk, diabetes, and racial interactions with each productive activity in year 1 and the Teng 3MS in year 11. In our working model, working and its racial interaction were not significant. Older Black vs. White adults scored -4.4 points lower; women, a high school degree or some post-secondary education, and former vs. never smokers related to higher; and each additional year of age and diabetes related to lower 3MS scores. In our volunteering model, volunteering was not significant. Older Black vs. White adults scored -5.2 points lower; women, a high school degree or some post-secondary education, and former vs. never smokers related to higher; each additional year of age and diabetes related to lower; and older Black vs. White volunteers scored 2.0 points higher 3MS scores. While racial disparities in 3MS scores existed in both models, older Black volunteers were cognitively protected. Our policy implication is to invest in older adults’ volunteering as social determinant interventions to maintain cognitive functioning and reduce racial disparities.

